# Validation of the German eHealth impact questionnaire for online health information users affected by multiple sclerosis

**DOI:** 10.1186/s12911-022-01968-6

**Published:** 2022-08-16

**Authors:** Anna Sippel, Karin Riemann-Lorenz, Jana Pöttgen, Renate Wiedemann, Karin Drixler, Eva Maria Bitzer, Christine Holmberg, Susanne Lezius, Christoph Heesen

**Affiliations:** 1 Institute of Neuroimmunology and Multiple Sclerosis, University Medical Center Hamburg-Eppendorf, Martinistrasse 52, 20246 Hamburg, Germany; 2 University of Education Freiburg, Freiburg, Germany; 3 Institute of Social Medicine and Epidemiology, Brandenburg Medical School Theodor Fontane, Brandenburg an der Havel, Germany; 4 Faculty of Health Sciences Brandenburg, Brandenburg Medical School Theodor Fontane, Potsdam, Germany; 5 Institute of Medical Biometry and Epidemiology, University Medical Center Hamburg-Eppendorf, Hamburg, Germany; 6 Department of Neurology, University Medical Center Hamburg-Eppendorf, Hamburg, Germany

**Keywords:** eHealth, Empowerment, Patient information, Psychometrics, Factor analysis, Multiple sclerosis

## Abstract

**Background:**

Persons with multiple sclerosis (MS) are confronted by an overwhelming amount of online health information, which can be valuable but also vary in quality and aim. Therefore, it is of great importance for developers and providers of eHealth information to understand its impact on the users. The eHealth Impact Questionnaire (eHIQ) has been developed in the United Kingdom to measure the potential effects of health and experimental information websites. This contains user’s general attitudes towards using the internet to gain health information and attitudes towards a specific health related website. The self-complete questionnaire is divided into two independently administered and scored parts: the 11-item eHIQ part 1 and the 26-item eHIQ part 2. This study aimed to validate the psychometric properties of the German version of the eHealth Impact Questionnaire (eHIQ-G).

**Methods:**

162 people with multiple sclerosis browsed one of two possible websites containing information on MS and completed an online survey. Internal consistency was assessed by Cronbach’s alpha and structural validity by Confirmatory Factor Analysis. Construct validity was examined by assessing correlations with the reference instruments eHealth Literacy Questionnaire and the General Self-Efficacy Scale measuring related, but dissimilar constructs. Moreover, we investigated the mean difference of the eHIQ-G score between the two websites. Data were analyzed using SPSS and AMOS software.

**Results:**

The eHIQ-G subscales showed high internal consistency with Cronbach’s alpha from 0.833 to 0.885. The 2-factor model of eHIQ part 1 achieved acceptable levels of goodness-of-fit indices, whereas the fit for the 3-factor model of eHIQ part 2 was poor and likewise for the alternative modified models. The correlations with the reference instruments were 0.08–0.62 and as expected. Older age was related with lower eHIQ part 1 score, whereas no significant effect was found for education on eHIQ part 1. Although not significant, the website ‘AMSEL’ reached higher mean scores on eHIQ part 2.

**Conclusions:**

The eHIQ-G has good internal consistency, and sufficient structural and construct validity. This instrument will facilitate the measurement of the potential impact of eHealth tools.

**Supplementary Information:**

The online version contains supplementary material available at 10.1186/s12911-022-01968-6.

## Background

With increased access to the Internet and technology, many easy access, and low cost opportunities for utilising electronic health (eHealth), or technology-delivered health information and services for the prevention and management of chronic diseases arise [1, 2]. Among chronically ill people, persons with multiple sclerosis (MS) are among the most frequent Internet users [3]. Thus, they are confronted by an overwhelming amount of online health information differing in quality and aim [3–5]. Methods to evaluate eHealth tools, such as mobile apps, online-portals for patients, and other Internet-based software or programs used to help patients monitor and manage their health have recently started to emerge [6]. A framework has been proposed by Allison et al. suggesting to evaluate website attributes, such as usability, content, web design, and functionality [7]. Currently, there is limited but increasing research looking at the potential impact of websites presenting individual patient experiences [8], since patient experiences are increasingly exchanged between patients and more frequently incorporated into health information websites [9]. It is crucial for developers and providers of online health information, especially experiential information, to understand its potential impact on knowledge, feeling of being supported, preparation for health decisions and behavioral outcomes on the users [10]. Therefore, instruments appropriate for assessing websites with different types of material, which can help to rate these dimensions from a user perspective are urgently needed [11].

In 2013, the eHealth Impact Questionnaire (eHIQ) was developed to measure self-reported impact of eHealth tools on the user [10] and validated as an English version in 2015 [12]. To our knowledge, only a validated Dutch and Hebrew version of the eHIQ exists [13, 14]. The eHIQ measures patient’s general attitudes towards using the Internet to obtain health information. Furthermore, it assesses impacts on patients after viewing a specific website containing different types of material (e.g., patients’ experiences, and scientific facts and figures). The impacts refer to 1) the extent to which patients gain confidence in discussing their health with others and the ability to identify with others; 2) the perceived ease of use, ease of understanding, trustworthiness and appropriateness of website content; and 3) the extent to which the patients better understand their health condition, feel reassured, and motivated to take action [10–12]. The eHIQ has already been used in several studies to evaluate eHealth tools, such as an online patient platform for communicating laboratory test results [15], a website to support families of burn-injured children [16], and a website providing narratives on prostate, breast and colorectal cancer [17]. The portal for laboratory test results achieved a high score on the usability scale of the eHIQ, but the portal could help the patients only slightly to take action in managing their own health [15]. The website providing burn-specific information was rated very positive and slightly better than the former portal [16]. The information on the website providing narratives was considered valuable and trustworthy by the majority of participants [17].

This study aimed to validate the eHIQ in a German population of eHealth users with MS by using confirmatory factor analysis and comparing the ratings for two websites of different complexity.

## Methods

### Recruitment and procedure

As we intended to use the scale in a clinical setting [18], participants were German-speaking persons with MS or people with suspected MS who were aged ≥ 18 years, and who had access to the Internet. The necessary sample size for validating questionnaires is contentious [19]. In accordance with our resources and the COSMIN Guidelines (Consensus-based Standards for the selection of health status Measurement Instruments) [20], we aimed to reach a sample size of 150.

Open recruitment took place from November 2019 to Mai 2020 through newsletters of the MS day hospital at the University Medical Center Hamburg-Eppendorf and as part of regular newsletters of four regional associations from the German Multiple Sclerosis Society (DMSG): Hamburg, Baden-Württemberg, Schleswig–Holstein, and Lower Saxony.

Persons with MS were invited to access an anonymous online survey by clicking on an electronic link. After reading the patient information, and giving informed consent online, patients were asked to fill out the following measures: the eHealth Literacy Questionnaire (eHLQ), eHIQ-part 1 and the General Self-Efficacy Scale (GSE). Afterwards, participants were directed to spend at least 10 minutes browsing either the section ‘living with MS’ of the website of the DMSG Baden-Württemberg, called AMSEL [21] or the whole website of the DMSG Hamburg [22]. In addition to factual information, the AMSEL website also contained explanatory films about living with MS from patients and health professionals. The DMSG Hamburg website contained only factual information at the time of the study. The websites were chosen to test if there is variation in the items of the eHIQ-G part 2 when rating websites with different types of health information such as facts, figures and personal experiences. After browsing one of the websites, patients had to return to the online survey and answer the eHIQ-part 2, and demographic as well as MS-related questions.

### Measures

The eHIQ is divided into two parts. The 11-item eHIQ-part 1 must be completed before accessing the website to be evaluated. It consists of two subscales (1) attitudes towards online health information and (2) attitudes towards sharing health experiences online. The 26-item eHIQ-part 2 measures the impact of using a specific health-related website on three subscales: (1) confidence and identification, (2) information and presentation, and (3) understanding and motivation. Response options range from 1 (‘strongly disagree’) to 5 (‘strongly agree’). The eHIQ part 2 must be administered after accessing the website to be evaluated. The scores were converted to a 0–100 metric. The total eHIQ score for part 1 and 2 was calculated as the sum of all subscale scores, divided by the number of subscales. Higher scores correspond with more positive responses [10, 12]. The translation of the eHIQ into German was performed in the context of a medical dissertation [23]. The translation was carried out according to the TRAPD (Translation, Review, Adjudication, Pretesting, and Documentation) team translation model in accordance with the Cross-Cultural Survey Guidelines [24]. Three staff members of the Institute of General Practice Göttingen produced three full translations of the eHIQ [23]. In the review phase, a team of five staff members of the same institute agreed on a single common translation. The translation of the individual questions either corresponded to one of the available translation suggestions or represented a new variant. This new version was submitted for a backward translation to a translator who had not been involved in any of the previous steps and was not familiar with the original English version of the eHIQ. The comparison of the original questionnaire with the backward translation led to further changes in the German translation resulting in a preliminary version of the eHIQ-G. Afterwards, the eHIQ-G was pretested in a convenience sample of 25 participants. The German version of the eHIQ can be found in the dissertation [23].

The eHLQ is a validated measure of eHealth literacy in English and Danish language covering user interaction with a given eHealth system and the user’s experience of engaging with it [25]. The eHLQ is valuable for the characterization and understanding of digital health literacy in a broad range of target groups. It contains 35 items in seven domains: (1) using technology to process health information, (2) understanding of health concepts and language, (3) ability to actively engage with digital services, (4) feel safe and in control, (5) motivated to engage with digital services, (6) access to digital services that work, and (7) digital services that suit individual needs [25]. Response options for all items range from 1 (strongly disagree) to 4 (strongly agree). The subscale scores are calculated summing up the scores of each item and dividing it by the number of items [25]. We used the eHLQ from the German eHLQ-validation study after back- and forward translation and a qualitative pre-test. The study has not yet been published.

The 10-item GSE scale was developed and validated to assess a general sense of perceived self-efficacy. Responses are made on a 4-point scale from 1 (not at all true) to 4 (exactly true). The total score is calculated by summing up all item scores. The total score ranges from 10 to 40, with a higher score indicating more self-efficacy [26].

Demographic data such as sex, age, educational level, and highest professional qualification were collected as well as MS-related information, e.g. the disease course, years since diagnosis, and the 9-item- ‘Patient Determined Disease Steps’ (PDDS), which asks for the patient-reported walking ability and disability (from 0 = normal to 8 = bedridden) [27].

### Data analysis

The analysis was performed in SPSS (version 25.0; IBM Corp.) and SPSS Amos (version 26.0; IBM Corp.) software. All analyses were carried out on complete cases. For sample description, continuous variables are described using mean and standard deviation (SD), and categorical items are presented as counts and percentages. To examine the internal consistency reliability of the five subscales, Cronbach’s alpha (α) was estimated. A Cronbach’s alpha value of > 0.7 was considered adequate [20].

Confirmatory factor analysis (CFA) was applied to investigate construct validity. The Full Information Maximum Likelihood estimation was used to estimate model parameters and to examine goodness-of-fit of all the CFA models with: the Root Mean Square Error of Approximation (RMSEA) ≤ 0.06, Standardized Root Mean Square (SRMR) ≤ 0.08, Tucker-Lewis-Index (TLI) ≥ 0.95, and Comparative Fit Index (CFI) ≥ 0.95 judged as adequate. Additionally, the minimum discrepancy (chi-square) per degree of freedom (CMIN/DF) ≤ 3 rule was also used [28, 29]. For inadequate model fit of the eHIQ-G, modification indexes were assessed [30] and an exploratory factor analysis (EFA) using Oblimin rotation and principal component analysis [31] was run to investigate an alternative to the original structure.

Moreover, convergent validity was assessed by testing hypotheses about expected relationships with eHLQ and GSE by calculating Pearson correlation coefficients. Correlations with instruments measuring related, but dissimilar constructs (eHLQ, GSE) should be 0.30–0.50 [20]. Convergent validity was considered adequate if at least 75% of the correlations were as expected. P values less than 0.05 are interpreted as statistically significant.

Hypothesis 1: Particular subscales of the eHIQ-G correlate with subscales of the eHLQ and with the GSE, which measure related, but dissimilar constructs such as the user’s interaction and experience with a given eHealth tool [13] and the perceived self-efficacy.

- The eHIQ-G part 1 subscale (1) ‘attitudes towards online health information’ correlates positively with the eHLQ subscales (1), (3), (5), (6), and (7).
- The eHIQ-G part 1 subscale (2) ‘attitudes towards sharing health experiences online’ correlates positively with the eHLQ subscales (1), (3), (5), (6), and (7).
- The eHIQ-G part 2 subscale (1) ‘confidence and identification’ correlates positively with the eHLQ subscales (5) and (7).
- The eHIQ-G part 2 subscale (2) ‘information and presentation’ correlates positively with the eHLQ subscale (2) and (4).
- The eHIQ-G part 2 subscale (3) ‘understanding and motivation’ correlates positively with the eHLQ subscales (2) and (5) as well as with the GSE score.
- The differences of the eHIQ-G according to characteristics of the participants were compared using t test, analysis of variance (ANOVA) and analysis of covariance (ANCOVA) to demonstrate convergent and discriminant validity.

Hypothesis 2: Higher educational levels predict higher scores on the eHIQ part 1 as persons with lower education seek health information online less likely [32].

Hypothesis 3: Younger persons are more likely to search for health-related information on the Internet [32]. Therefore, younger age predicts higher scores on the eHIQ part 1.

Hypothesis 4. The website of AMSEL, which contains factual and experiential information gets a higher sum index score on eHIQ part 2 than the website of DMSG Hamburg, which shows only factual information.

## Results

### Sample characteristics

**Fig. 1:**
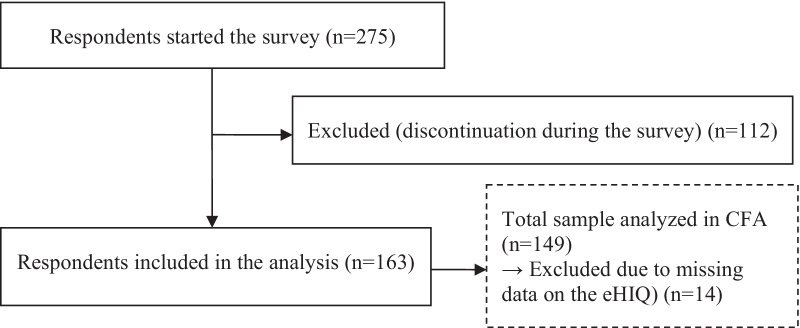
Participant flow chart

163 persons with MS were enrolled in the study (Table 1). The response rate (59.3%) was calculated as the number of returned questionnaires (N = 163) divided by the total sample who logged in the survey (N = 275) (Fig. 1).

**Table 1:** Demographic and clinical characteristics of the study sample (N = 163)

Characteristic	Value
Sex, female, n (%)	115 (71.0)
Age in years, mean (SD)	51 (11.0)
Education level, n (%)	
Primary degree (9 grades)	8 (4.9)
Secondary degree (10 grades)	48 (29.6)
High school degree (12/13 grades)	106 (65.4)
MS diagnosis, n (%)	
Suspicion of MS	8 (4.9)
MS diagnosis	154 (95.1)
Years with MS since diagnosis, mean (SD)^1^	14 (9.8)
MS type, n (%)	
Relapsing–remitting MS	87 (53.7)
Secondary-progressive MS	41 (25.3)
Primary-progressive MS	23 (14.2)
Unclear	11 (6.8)
Patient determined disease steps (PDDS), mean (SD)	3.1 (2.2)
Website browsed, n (%)	
DMSG Hamburg	106 (65.4)
Usage of this website prior to this study	
Regular use (> 2 times over the past year)	28 (26.4)
Occasional use (1–2 over the past year)	26 (24.5)
Infrequent use (more than a year ago)	28 (26.4)
No usage	24 (22.6)
AMSEL	56 (34.6)
Usage of this website prior to this study	
Regular use (> 2 times over the past year)	19 (33.9)
Occasional use (1–2 over the past year)	14 (25.0)
Infrequent use (more than a year ago)	12 (21.4)
No usage	11 (19.6)

^1^N = 151

Most of the participants were female (71.0%). The mean age was 51 years. The level of education was high with 65.4% reporting a high school degree. Nearly all participants had a definite MS diagnosis, while 4.9% were suspected having MS. 54% of those with definite MS had a relapsing–remitting MS course. The patients had lived with the disease on average for 14 years and had on average a PDDS of 3.1 (gait disability). Most participants (65.4%) decided to spend time on the website of the DMSG Hamburg, while 34.6% browsed the website of the AMSEL. Regarding the use of those websites before participation in this study, 77.4% had already used the DMSG Hamburg website, while 80.4% had already used the AMSEL website.

### Internal reliability

All subscales showed good internal consistency with Cronbach’s α from 0.833 to 0.885 (Table 2). Overall internal consistency for the entire eHIQ-G was 0.926.

**Table 2:** Parameter estimates of the confirmatory factor analysis (N = 149)

Factors	Items	SE^2^	CR^3^	*p*	β^4^	R^2 5^	α ^6^
*eHIQ 1*							
1) Attitudes towards online health information							0.833
	eHIQ1 item 1	0.10	8.10	< 0.001	0.74	0.54	
	eHIQ1 item 2	0.11	7.10	< 0.001	0.66	0.44	
	eHIQ1 item 3	0.11	7.14	< 0.001	0.66	0.44	
	eHIQ1 item 4	0.12	8.11	< 0.001	0.71	0.51	
	eHIQ1 item 5^1^				0.73	0.53	
2) Attitudes towards sharing health information							0.867
	eHIQ1 item 6	0.09	7.74	< 0.001	0.63	0.40	
	eHIQ1 item 7	0.09	8.88	< 0.001	0.72	0.52	
	eHIQ1 item 8	0.10	7.83	< 0.001	0.64	0.41	
	eHIQ1 item 9	0.10	9.14	< 0.001	0.71	0.51	
	eHIQ1 item 10	0.09	11.19	< 0.001	0.86	0.74	
	eHIQ1 item 11^1^				0.79	0.63	
*eHIQ 2*							
1) Confidence and identification							0.883
	eHIQ2 item 10	0.13	5.27	< 0.001	0.47	0.22	
	eHIQ2 item 11	0.13	7.75	< 0.001	0.72	0.52	
	eHIQ2 item 14	0.13	7.70	< 0.001	0.69	0.48	
	eHIQ2 item 15	0.13	8.10	< 0.001	0.73	0.54	
	eHIQ2 item 17	0.10	8.81	< 0.001	0.83	0.68	
	eHIQ2 item 18	0.12	7.11	< 0.001	0.64	0.40	
	eHIQ2 item 19	0.12	8.04	< 0.001	0.72	0.52	
	eHIQ2 item 20	0.13	7.82	< 0.001	0.69	0.47	
	eHIQ2 item 23^1^				0.67	0.45	
2) Information and presentation							0.838
	eHIQ2 item 3	0.17	6.20	< 0.001	0.60	0.36	
	eHIQ2 item 5	0.16	6.57	< 0.001	0.68	0.46	
	eHIQ2 item 6	0.12	6.70	< 0.001	0.65	0.42	
	eHIQ2 item 9	0.12	7.49	< 0.001	0.75	0.56	
	eHIQ2 item 12	0.15	6.33	< 0.001	0.66	0.43	
	eHIQ2 item 24	0.15	6.51	< 0.001	0.66	0.44	
	eHIQ2 item 25	0.16	4.88	< 0.001	0.46	0.21	
	eHIQ2 item 26^1^				0.64	0.40	
3) Understanding and motivation							0.885
	eHIQ2 item 1	0.12	8.94	< 0.001	0.75	0.56	
	eHIQ2 item 2	0.11	7.49	< 0.001	0.64	0.40	
	eHIQ2 item 4	0.10	8.29	< 0.001	0.70	0.49	
	eHIQ2 item 7	0.11	8.03	< 0.001	0.67	0.45	
	eHIQ2 item 8	0.13	7.26	< 0.001	0.61	0.38	
	eHIQ2 item 13	0.13	7.08	< 0.001	0.59	0.35	
	eHIQ2 item 16	0.11	8.27	< 0.001	0.69	0.48	
	eHIQ2 item 21	0.11	9.05	< 0.001	0.74	0.55	
	eHIQ2 item 22^1^				0.73	0.54	

^1^ = This regression weight was fixed at 1.000, not estimated

^2^*SE*  Standard error

^3^*CR*  Critical ratio

^4^β = Standardized regression estimate

^5^R^2^ = Squared multiple correlations estimate

^6^α = Cronbach’s alpha

### Structural validity

**Fig. 2:**
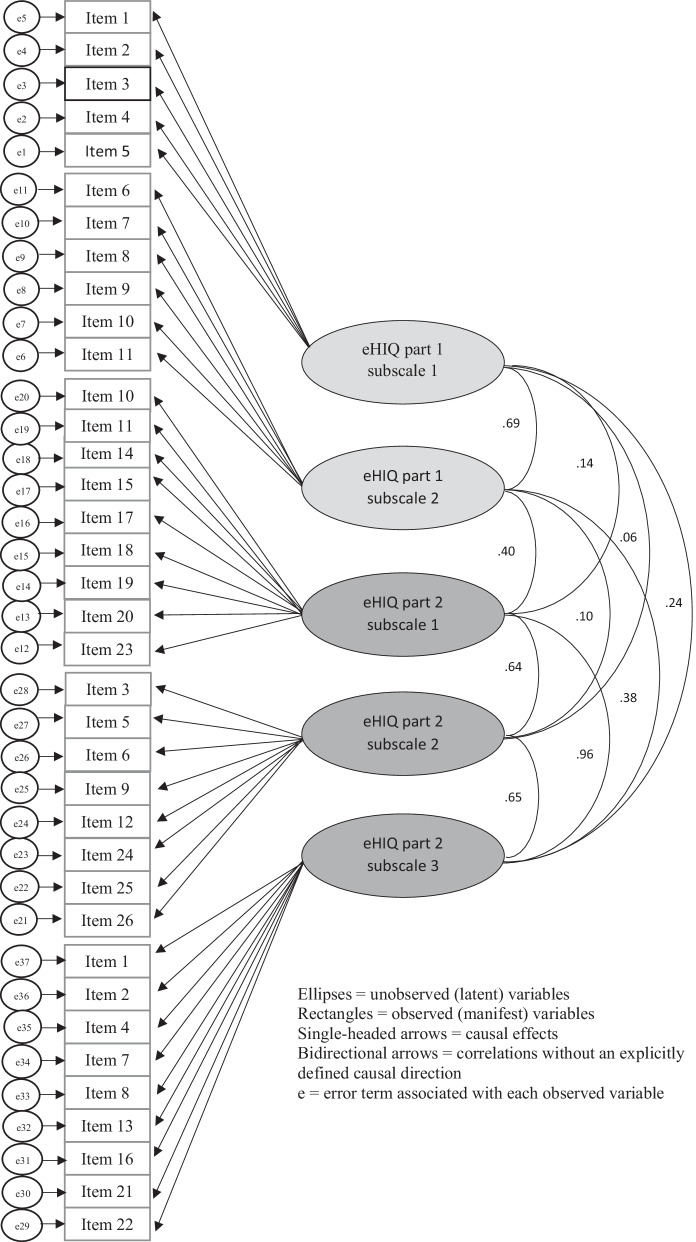
Path model showing relationship among latent variables and manifest variables of the original eHIQ-G

The CFA was run on 149 complete cases that were only participants for whom we had no missing data on the eHIQ-G scale (Fig. 1). The analysis was performed on the five factors and 37 items (Fig. 2).

All items had a standardized factor load (β) of ≥ 0.50 and a critical ratio value (C.R.) of ≥ 1.96 (*p* < 0.05) indicating a good model identification [33] except for eHIQ part 2 item 10 and 25 (Table 2). Results of the CFA on 149 complete cases for the whole eHIQ-G including part 1 and 2 suggested poor fit to the data (RMSEA = 0.09, SRMR = 0.09, CFI = 0.75, TLI = 0.73). Solely CMIN/DF = 2.26 showed an acceptable fit.

Additionally, two CFAs were run separately for each part of the eHIQ-G. The goodness-of-fit indices of the eHIQ-G part 1 model were as follows: RMSEA = 0.12, SRMR = 0.07, CFI = 0.91, TLI = 0.88, CMIN/DF = 2.63. All the fit indices ranged from satisfactory to poor. An EFA was run to investigate an alternative to the original structure of the eHIQ-G part 1. The Kaiser–Meyer–Olkin (KMO) measure verified the sampling adequacy for the analysis, KMO = 0.87. The EFA resulted in the same two factors with the same items (predictors) as the original model.

The fit for the eHIQ-G part 2 model was poor (RMSEA = 0.12, SRMR = 0.10, CFI = 0.74, TLI = 0.71, CMIN/DF = 3.00). Inspecting the modification index output (Additional file 1), which showed covariances that could be incorporated into a re-specified model to obtain superior goodness-of-fit, covariances were found between items 14 and 15 as well as between items 20 and 23. After removing items 15, 20 and incorporating item 10 into the subscale eHIQ 2.3 the fit for the alternative model (Additional file 2) was better, but still not acceptable (RMSEA = 0.12, SRMR = 0.10, CFI = 0.77, TLI = 0.75, CMIN/DF = 2.75). The EFA for the eHIQ-G part 2 suggested five factors, which were not clearly interpretable and had many items that had double loadings. Furthermore, we applied the alternative Dutch 3-factor structure [13] (Additional file 3) in our CFA and resulted in better, but also unacceptable fit indices (RMSEA = 0.11, SRMR = 0.10, CFI = 0.76, TLI = 0.74, CMIN/DF = 2.81). The path diagrams (Fig. 2, Additional files 2 and 3) showed partly high correlations between the subscales (latent factors). As this was already found by the English validation study [12], we have decided to stay with the original 3-factor model of the English eHIQ part 2.

### Convergent and discriminant validity

Descriptive data for the GSE, eHLQ subscales, and eHIQ-G part 1 are shown in Table 3. Participants considered their knowledge and skills related to eHealth literacy to be moderate. The mean score for participants’ general attitudes towards using the Internet to access health information and their ease with using online (experiential) information for learning and gaining support (eHIQ-G part 1) was medium. Despite the concentration of eHIQ-G scores at the positive end of the construct, the distributions were sufficiently symmetric. The eHLQ subscales (with data of the subscale 1 concentrating on the positive end, and eHLQ subscale 4 on the negative end) as well as the GSE were approximately normally distributed.

**Table 3:** Descriptive data of outcome measures

Measures	N	Mean (range)	SD
GSE score (mean, range)	161	29.42 (17–40)	5.2
eHLQ scores			
1. Using technology to process health information	158	2.66 (1–4)	0.6
2. Understanding of health concepts and language	157	3.00 (2–4)	0.5
3. Ability to actively engage with digital services	153	2.96 (1–4)	0.6
4. Feel safe and in control	152	2.56 (1–4)	0.6
5. Motivated to engage with digital services	158	2.51 (1–4)	0.6
6. Access to digital services that work	156	2.32 (1–4)	0.5
7. Digital services that suit individual needs	157	2.32 (1–4)	0.5
eHIQ scores			
eHIQ part 1 sum index score	158	53.69 (0–100)	20.6
1.1 Attitudes towards online health information	161	51.58 (0–100)	22.4
1.2 attItudes towards sharing health experiences online	159	55.74 (0–100)	23.6

**Fig. 3:**
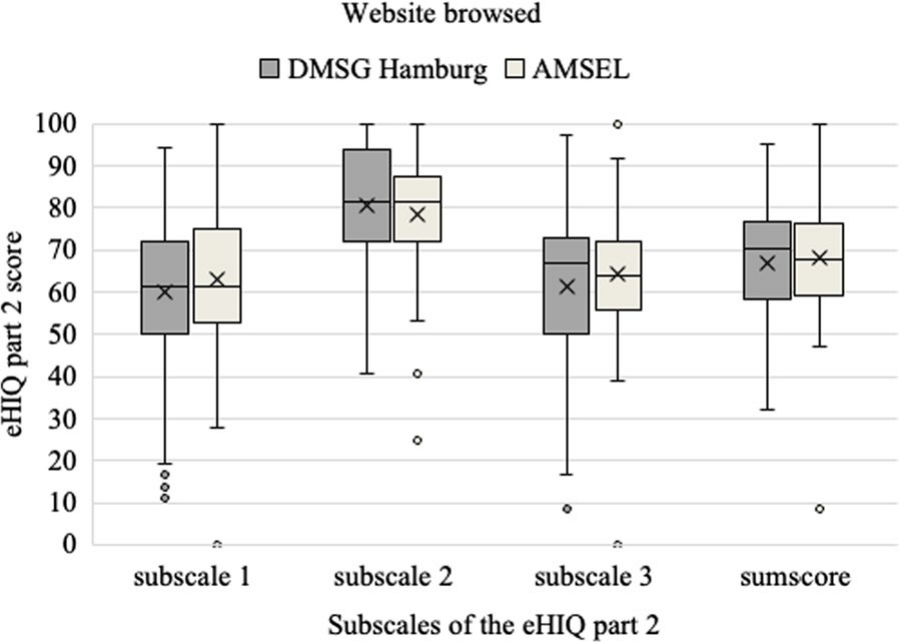
Comparison of the eHIQ-G part 2 score for websites of DMSG Hamburg and AMSEL (N = 151)

Figure 3 illustrates the eHIQ-G part 2 subscale scores and the sum index score of each website. Data were displayed in the boxplot by the minimum, the maximum, the median, the mean, the first, and third quartiles, and the outliers (dots). Both websites gained moderate sum index scores on the eHIQ-G part 2. The websites achieved the highest score in subscale 2.2, which reflects users’ trust and suitability of the website content. Participants’ confidence to discuss their health condition with others after browsing the websites and their ability to identify with the websites (eHIQ-G 2.1) was moderate. The score on subscale 2.3, which reflects the understanding and learning about relevant health information and motivation to act was moderate as well. The t test indicated no significant difference in mean in eHIQ-G part 2 sum index score between the websites of DMSG Hamburg and AMSEL (t 149 = − 0.45, *p* = 0.35, mean difference = − 0.40, 95% CI = − 6.08–4.04). No significant differences in means of the subscale 2.1 (t 154 = − 1.00, *p* = 0.32, mean difference = − 3.01, 95% CI = − 9.08–3.00), subscale 2.2 (t 156 = 0.95, *p* = 0.35, mean difference = 2.35, 95% CI = − 2.56–7.25) and subscale 2.3 (t 129.55 = − 1.08, *p* = 0.28, mean difference = − 3.05, 95% CI = − 8.96–2.87) between DMSG Hamburg and AMSEL could be proven. However, descriptively the average mean eHIQ-G part 2 sum index score as well as in subscales 2.1, and 2.3 for the AMSEL website were descriptively higher than the average sum index score for DMSG Hamburg.

Moreover, relationships (Pearson’s correlation coefficients) between eHIQ-G subscales and the selected reference measures were examined to assess construct validity. Results confirmed our expectations that almost all scales are significantly related: a large positive correlation was found between the eHIQ-G (1.1) and eHLQ (1), and (5) as well as a positive moderate to small correlation between the eHIQ-G (1.2) and eHLQ (3), (6), and (7). Correlations between eHIQ-G (2.1) and eHLQ (5), and (7) were small. There were either small (r < 0.20) or no significant (*p* = 0.17) correlations between eHIQ-G (2.2) and eHLQ (2), and (4). Only a positive small correlation was found between eHIQ-G (2.3) and eHLQ (5). The eHIQ-G (2.3) did not correlate significantly (*p* = 0.32) with the GSE score (Table 4).

**Table 4:** Pearson correlations among eHIQ-G, eHLQ and GSE

	eHIQ 1.1	eHIQ 1.2	eHIQ 1^3^	eHIQ 2.1	eHIQ 2.2	eHIQ 2.3	eHIQ 2^3^
eHLQ 1	0.57^2^	0.52^2^	0.62^2^				
eHLQ 2					0.28^2^	0.19^1^	0.22^2^
eHLQ 3	0.32^2^	0.28^2^	0.34^2^				
eHLQ 4					0.12		
eHLQ 5	0.57^2^	0.47^2^	0.59^2^	0.25^2^		0.30^2^	
eHLQ 6	0.21^1^	0.15	0.20^1^				
eHLQ 7	0.37^2^	0.30^2^	0.37^2^	0.26^2^			
GSE						0.08	

^1^ = Correlation is significant at the 0.05 level (2-tailed)

^2^ = Correlation is significant at the 0.01 level (2-tailed)

^3^ = Sum index score

N = 152–161 participants

Older age was related with lower eHIQ-G part 1 sum index score per 5 years [F (1, 154) = 10.00, B = − 2.29, *p* < 0.001, partial Eta Squared = 0.06]. On the other hand, multilevel ANCOVA did not reveal influence of education on the eHIQ-G part 1 [F (2, 154) = 1.14, *p* = 0.32, partial Eta Squared = 0.02]. Thus, discriminant validity could be shown at least partially.

## Discussion

Web-based health information and interventions are a substantially emerging area of providing treatment for various conditions or supporting self-management [34–36] which has been even accelerated in the COVID-19 pandemic [37]. However, critical appraisal of eHealth information is a challenge and the real impact on health behaviour and health state is a matter of emerging research [11]. As a valid and reliable instrument was lacking in Germany, this study aimed to examine the structural validity, internal consistency, and construct validity of the eHIQ-G in a sample of persons with MS. The results show that the German eHIQ has sufficient structural validity, internal consistency, and construct validity as well as conditionally sufficient structural validity.

The CFA analyses showed satisfactory fit indices for the original eHIQ-G part 1. Similar results have been found for the Dutch version of the original model. The fit for the eHIQ-G part 2 was poor and likewise for the alternative 3-factor model. Neijenhuijs et al. reported bad fit indices for the original model of the Dutch eHIQ part 2, too. An alternative 3-factor structure was investigated and resulted in a good model fit [13]. No model-fit-indices were reported for the Hebrew version [14]. We have investigated an alternative model based on the modification index output as well as on the alternative Dutch factor structure which resulted in better, but still unacceptable fit indices. The developers of the eHIQ found that 12 items of the eHIQ part 2 were loading on more than one subscale [12]. However, items were allocated to the subscale on which they loaded most highly and made conceptually sense [12]. To have comparable results with other countries and to avoid a shift in the model’s meaning from a theoretical standpoint, we decided to stick to the original factor structure of eHIQ part 2.

Evidence for sufficient internal consistency of the eHIQ-G subscales was indicated by a Cronbach’s alpha of > 0.70 [20]. The internal consistency found in this study was comparable to the internal consistency found in previous studies [12–14].

As no validated reference inventory in German language exists, which measures the self-reported impact of eHealth tools on the user, we selected related, but dissimilar construct measurement tools such as the eHLQ [25] and the GSE [26]. Predominantly all expectations towards hypothesis one were confirmed. However, many of the correlations were small. Our results are difficult to compare with the results of the other validation studies [12, 13], since others did use other reference measures. In these studies authors report finding small to acceptable correlations for convergent and divergent validity [12, 13]. We were not able to use these questionnaires as they were neither available in German nor validated. We could only partially confirm discriminant validity (hypotheses 3–4): older age was related with lower eHIQ-G part 1 score, whereas no significant effect was found for education on eHIQ-G part 1. Kelly et al. found no significant difference for age [12]. No significant difference was found for the browsed websites among all subscale scores of the eHIQ-G part 2 (hypothesis 4). This did not match our assumption that the AMSEL being richer in different types of information, e.g., providing MS patients with videos on how to manage their daily lives, would get higher scores on the average mean eHIQ-G part 2 sum index score.

### Limitations

This study has several limitations: as with most web–based studies, a selection bias related to recruitment of volunteer participants is also present in our study [38]. Almost two-thirds of the participants were highly educated and therefore, evidence for low-educated persons is limited. In fact, individuals with low level of education are less likely to participate in health examination surveys [39, 40] and focussed efforts are necessary to include low-educated persons in evaluations of eHealth tools. Second, although the sample size was adequate according to KMO, other sources [31] say that more data are needed to perform CFA. In this study, only persons with MS were chosen to represent the target population of the eHIQ-G. Like other people with especially chronic conditions, persons with MS increasingly search for health information on the Internet [41]. Among chronically ill people, they are among the most frequent Internet users as they are young, now mostly digital natives, mostly not substantially impaired in the early phase of the disease [42, 43]. Therefore, persons with MS represent a prototypic, ideal eHealth population. However, other patient groups should be included to test whether the eHIQ-G is applicable to a range of different conditions [11]. Besides, we could show discriminant validity only based on differences of the eHIQ-G scale performance at different ages. We did not assess discriminant validity with reference inventories to be regarded as measuring distinct constructs [44], as we were lacking scales measuring related constructs based on theory or prior empirical observations. Finally, we used an anonymous survey for pragmatic and data protection reasons while collecting data. Therefore, we did not test for test–retest reliability to examine the consistency over time.

## Conclusion

The eHIQ-G is a reliable and valid inventory with acceptable psychometric properties assessed in a group of patients with MS. However, the subscales of the eHIQ-G part 2 and the corresponding items have conceptional limitations. Lastly, this is the first study evaluating the German version of eHIQ in MS patients and suggesting a 37-item model, which is possibly able to describe different important aspects of health websites with various styles of information from a user perspective. The eHIQ-G is proposed for usage in future studies on the impact of websites containing various styles of health information. The eHIQ-G can be used to reflect on user’s general attitudes towards health-related websites (eHIQ part 1) and on the website’s design, credibility, reputation, and the possible impacts (eHIQ part 2). Therefore, it can be used to test and improve websites with health information. Additionally, the inventory is promoting research and comparison among different websites, since it has already been translated in several languages and validated in several groups. Despite the limitations of the eHIQ-G part 2, we recommend considering the eHIQ-G in future studies assessing the impact of eHealth tools on its users.

## Supplementary Information

**Additional file 1:** Modification Indices—Residual Covariances between eHIQ-G part 2 items.

**Additional file 2:** Path model of factors and indicators of the alternative model of the eHIQ-G part 2.

**Additional file 3:** Path model of factors and indicators of the Dutch alternative model of the eHIQ-G part 2.

## Data Availability

The data are not publicly available due to privacy issues but will be available from the corresponding author on reasonable request.

## Abbreviations

ANCOVA: Analysis of covariance
ANOVA: Analysis of variance
CFA: Confirmatory factor analysis
CFI: Comparative fit index
CMIN/DF: Minimum discrepancy (chi-square) per degree of freedom
DMSG: German multiple sclerosis society
EFA: Exploratory factor analysis
eHIQ: EHealth impact questionnaire
eHLQ: EHealth literacy questionnaire
GSE: General self-efficacy
KMO: Kaiser–Meyer–Olkin
MS: Multiple sclerosis
PDDS: Patient determined disease steps
PEx: Patient experiences
RMSEA: Root mean square error of approximation
SRMR: Standardized root mean square
TLI: Tucker-Lewis-index
