# Supplementary material for: Validation of the German eHealth impact questionnaire for online health information users affected by multiple sclerosis
Source: BMC Med Inform Decis Mak. 2022 Aug 16;22:219. doi: 10.1186/s12911-022-01968-6 (PMC9380659; doi:10.1186/s12911-022-01968-6)
Supplement: Supplementary file 1 — Additional file 1: Modification Indices—Residual Covariances between eHIQ-G part 2 items. [file 12911_2022_1968_MOESM1_ESM.docx]

Additional file 1. Modification Indices – Residual Covariances between eHIQ-G part 2 items

| **Residual covariances between eHIQ-G part 2 items** | | | **Modification index** | **Par Change** |
| --- | --- | --- | --- | --- |
| e36 | <--> | information_and_presentation | 18.874 | .131 |
| e36 | <--> | e37 | 11.288 | .145 |
| e35 | <--> | e37 | 6.338 | .090 |
| e34 | <--> | Confidence_and_identification | 5.600 | -.052 |
| e34 | <--> | Understanding_and_motivation | 6.195 | .053 |
| e33 | <--> | e37 | 5.779 | .130 |
| e33 | <--> | e34 | 8.073 | .153 |
| e32 | <--> | information_and_presentation | 7.615 | .105 |
| e31 | <--> | information_and_presentation | 6.049 | -.071 |
| e31 | <--> | e37 | 8.256 | -.119 |
| e31 | <--> | e34 | 14.271 | .155 |
| e31 | <--> | e32 | 4.445 | -.112 |
| e30 | <--> | e36 | 4.253 | -.085 |
| e29 | <--> | e36 | 5.385 | -.097 |
| e29 | <--> | e35 | 4.272 | -.072 |
| e29 | <--> | e30 | 12.464 | .139 |
| e28 | <--> | e37 | 12.045 | .169 |
| e28 | <--> | e36 | 16.275 | .200 |
| e27 | <--> | information_and_presentation | 11.311 | -.092 |
| e27 | <--> | Understanding_and_motivation | 5.978 | .051 |
| e26 | <--> | information_and_presentation | 5.795 | .055 |
| e26 | <--> | e36 | 6.412 | .086 |
| e26 | <--> | e29 | 4.638 | -.069 |
| e25 | <--> | e37 | 4.942 | -.068 |
| e25 | <--> | e26 | 18.956 | .103 |
| e24 | <--> | e32 | 9.209 | .159 |
| e24 | <--> | e30 | 4.646 | .084 |
| e23 | <--> | information_and_presentation | 4.726 | -.061 |
| e23 | <--> | Confidence_and_identification | 10.587 | .070 |
| e23 | <--> | e29 | 7.722 | .109 |
| e22 | <--> | Confidence_and_identification | 4.242 | -.056 |
| e22 | <--> | e36 | 8.457 | .154 |
| e22 | <--> | e28 | 30.636 | .328 |
| e21 | <--> | e25 | 6.712 | .081 |
| e21 | <--> | e24 | 8.037 | -.119 |
| e20 | <--> | Confidence_and_identification | 5.662 | -.071 |
| e20 | <--> | Attitudes_towards_online_health_information | 4.704 | .136 |
| e20 | <--> | Understanding_and_motivation | 4.456 | .062 |
| e20 | <--> | e33 | 15.048 | .281 |
| e20 | <--> | e22 | 4.538 | -.147 |
| e19 | <--> | information_and_presentation | 23.202 | .144 |
| e19 | <--> | Confidence_and_identification | 4.950 | -.049 |
| e19 | <--> | e37 | 6.451 | .108 |
| e19 | <--> | e36 | 11.656 | .148 |
| e19 | <--> | e30 | 6.173 | -.101 |
| e19 | <--> | e29 | 11.902 | -.142 |
| e19 | <--> | e28 | 13.856 | .182 |
| e19 | <--> | e20 | 7.926 | .160 |
| e18 | <--> | information_and_presentation | 9.762 | -.104 |
| e18 | <--> | e31 | 14.287 | .176 |
| e17 | <--> | Confidence_and_identification | 8.358 | .064 |
| e17 | <--> | Understanding_and_motivation | 9.221 | -.067 |
| e17 | <--> | e18 | 45.874 | .327 |
| e16 | <--> | Confidence_and_identification | 7.139 | -.037 |
| e16 | <--> | Understanding_and_motivation | 5.090 | .031 |
| e16 | <--> | e36 | 5.449 | .065 |
| e16 | <--> | e35 | 11.820 | .080 |
| e16 | <--> | e29 | 6.547 | -.068 |
| e16 | <--> | e19 | 7.374 | .074 |
| e16 | <--> | e18 | 5.527 | -.072 |
| e15 | <--> | information_and_presentation | 7.228 | .078 |
| e15 | <--> | Confidence_and_identification | 6.491 | -.055 |
| e15 | <--> | e35 | 14.859 | -.135 |
| e15 | <--> | e30 | 4.943 | .088 |
| e15 | <--> | e29 | 30.070 | .220 |
| e15 | <--> | e24 | 8.072 | .113 |
| e15 | <--> | e18 | 5.499 | -.109 |
| e15 | <--> | e17 | 5.703 | -.100 |
| e14 | <--> | Confidence_and_identification | 6.413 | .054 |
| e14 | <--> | e37 | 4.507 | -.087 |
| e14 | <--> | e33 | 5.142 | -.119 |
| e14 | <--> | e20 | 4.208 | -.113 |
| e13 | <--> | information_and_presentation | 13.523 | -.127 |
| e13 | <--> | e36 | 8.464 | -.146 |
| e13 | <--> | e30 | 10.734 | .155 |
| e13 | <--> | e29 | 13.595 | .176 |
| e13 | <--> | e28 | 6.887 | -.149 |
| e13 | <--> | e26 | 5.158 | -.088 |
| e13 | <--> | e19 | 19.853 | -.221 |
| e13 | <--> | e16 | 4.999 | -.071 |
| e13 | <--> | e14 | 18.220 | .204 |
| e12 | <--> | e36 | 9.381 | -.154 |
| e12 | <--> | e30 | 6.748 | .123 |
| e12 | <--> | e29 | 20.963 | .219 |
| e12 | <--> | e28 | 4.261 | -.117 |
| e12 | <--> | e23 | 6.168 | .118 |
| e12 | <--> | e21 | 5.493 | .120 |
| e12 | <--> | e20 | 4.846 | -.145 |
| e12 | <--> | e19 | 15.446 | -.195 |
| e12 | <--> | e16 | 6.344 | -.080 |
| e12 | <--> | e13 | 51.647 | .413 |
| e11 | <--> | information_and_presentation | 7.471 | .097 |
| e11 | <--> | e31 | 4.322 | -.103 |
| e11 | <--> | e29 | 4.088 | -.099 |
| e11 | <--> | e24 | 4.726 | .105 |
| e10 | <--> | e20 | 4.723 | -.143 |
| e10 | <--> | e11 | 18.449 | .251 |
| e9 | <--> | Attitudes_towards_online_health_information | 4.389 | -.141 |
| e8 | <--> | e11 | 5.506 | -.150 |
| e8 | <--> | e10 | 7.998 | -.176 |
| e7 | <--> | e26 | 5.924 | .084 |
| e7 | <--> | e23 | 4.677 | -.092 |
| e7 | <--> | e14 | 6.155 | -.106 |
| e7 | <--> | e9 | 4.228 | .129 |
| e6 | <--> | e30 | 4.548 | .104 |
| e6 | <--> | e29 | 5.055 | .111 |
| e6 | <--> | e13 | 5.819 | .143 |
| e5 | <--> | e9 | 11.192 | -.218 |
| e4 | <--> | e22 | 4.744 | -.132 |
| e4 | <--> | e20 | 14.483 | .252 |
| e4 | <--> | e15 | 6.296 | -.122 |
| e4 | <--> | e11 | 4.597 | .126 |
| e3 | <--> | information_and_presentation | 4.092 | -.072 |
| e3 | <--> | e8 | 4.660 | .139 |
| e2 | <--> | e26 | 4.146 | .089 |
| e2 | <--> | e4 | 8.485 | -.189 |
| e2 | <--> | e3 | 6.951 | .175 |
| e1 | <--> | e37 | 4.712 | -.122 |
| e1 | <--> | e13 | 7.302 | -.177 |
| e1 | <--> | e12 | 6.288 | -.164 |
| e1 | <--> | e3 | 4.395 | -.139 |
| e1 | <--> | e2 | 6.529 | .187 |
