# Supplementary material for: Validation of the German eHealth impact questionnaire for online health information users affected by multiple sclerosis
Source: BMC Med Inform Decis Mak. 2022 Aug 16;22:219. doi: 10.1186/s12911-022-01968-6 (PMC9380659; doi:10.1186/s12911-022-01968-6)
Supplement: Supplementary file 3 — Additional file 3: Path model of factors and indicators of the Dutch alternative model of the eHIQ-G part 2. [file 12911_2022_1968_MOESM3_ESM.docx]

**Path model showing relationship among latent factors and manifest indicators of the Dutch alternative model of the eHIQ-G part 2**

Ellipses = unobserved (latent) variables

Rectangles = observed (manifest) variables

Single-headed arrows = causal effects

Bidirectional arrows = correlations without an explicitly defined causal direction

e = error term associated with each observed variable

Additional file 3. Path model of factors and indicators of the Dutch alternative model of the eHIQ-G part 2.

.48

.72

.80

Item 18

Item 3

Item 17

Item 14

Item 19

Item 5

Item 23

Item 10

Item 26

Item 12

Item 25

Item 24

Item 9

Item 1

Item 2

Item 6

Item 22

Item 21

Item 16

Item 7

Item 8

Item 4

Item 13

Item 11

Item 15
